# A comparative assessment of the accuracy of electronic apex locator (Root ZX) in the presence of commonly used irrigating solutions

**DOI:** 10.4317/jced.51230

**Published:** 2014-02-01

**Authors:** Osama Khattak, Ebadullah Raidullah, Maria L. Francis

**Affiliations:** 1 Assistant Professor of Endodontics and Co Ordinator Dental Clinics, RAK College of Dental Scineces, Ras al Khaimah, UAE; 2 Junior Instructor, RAK College of Dental Sciences, Ras al Khaimah, UAE; 3 Intern dentist, RAK College of Dental Sciences, Ras al Khaimah, UAE

## Abstract

Objectives: This study aimed to evaluate the accuracy of Root ZX in determining working length in presence of normal saline, 0.2% chlorhexidine and 2.5% of sodium hypochlorite. Material and Methods: Sixty extracted, single rooted, single canal human teeth were used. Teeth were decoronated at CEJ and actual canal length determined. Then working length measurements were obtained with Root ZX in presence of normal saline 0.9%, 0.2% chlorhexidine and 2.5% NaOCl. The working length obtained with Root ZX were compared with actual canal length and subjected to statistical analysis. Results: No statistical significant difference was found between actual canal length and Root ZX measurements in presence of normal saline and 0.2% chlorhexidine. Highly statistical difference was found between actual canal length and Root ZX measurements in presence of 2.5% of NaOCl, however all the measurements were within the clinically acceptable range of ±0.5mm. Conclusion: The accuracy of EL measurement of Root ZX within±0.5 mm of AL was consistently high in the presence of 0.2% chlorhexidine, normal saline and 2.5% sodium hypochlorite. Clinical significance: This study signifies the efficacy of ROOT ZX (Third generation apex locator) as a dependable aid in endodontic working length.

** Key words:**Electronic apex locator, working length, root ZX accuracy, intracanal irrigating solutions.

## Introduction

The removal of all pulp tissue, necrotic material and microorganisms from the root canal is essential for endodontic success. On the other hand determination of an accurate working length is also a critical step in endodontic therapy (1). Therefore proper instrumentation upto the apical constriction or also called as the cemento-dentinal junction (2) as seen earlier is also one of the vital factor for a good prognosis. Traditionally, the point of termination for endodontic instrumentation and obturation has been determined by taking radiographs. But just how accurate is this radiographic measurement? For one thing, accuracy depends on the radiographic technique used. Forsberg, in Norway, demonstrated that paralleling technique was “significantly more reliable” than the bisecting-angle technique (3). A US Army group, however, found that the paralleling technique was absolutely accurate only 82% of the time (4) One study suggested that paralleling technique magnified actual tooth length by 5.4% (5). As Olson et al. pointed out, 82 to 89% accuracy is not 100%, so they recommended back-up methods such as tactile feel, moisture on the tip of a paper point, or electronic apex locators (4). Similar results and recommendations have been reported worldwide (6-11).

An in vivo histological study found that the most favourable histological conditions were when the instrumentation and obturation remained short of the apical constriction and that extruded gutta-percha and sealer always caused a severe inﬂammatory reaction despite the absence of pain (12). The problem clinicians face is how to accurately identify and prepare to this landmark – the ‘working length’ – and achieve maximum success.

The radiographs are definitely supportive for the instrumentation upto the apical constriction, but they can also prove deceptive due to improper angulation of the cone. The radiographic assessment technique is sensitive in both its exposure and interpretation (13). Also, the image obtained is a two-dimensional image of a three dimensional object. Therefore keeping the above limitations in mind an alternative for radiographic apex locator was very much needed. First electronic apex locator was introduced in 1918 by Custer et al. His ideas were later revisited by Suzuki in 1942 (14) and Sunada in 1962 (15) for the invention of the modern electronic apex locator apex. Thus the development of modern electronic apex locator has helped make the assessment of working length more accurate and predictable (16).

All apex locators function by using the human body to complete an electrical circuit. One side of the apex locator’s circuitry is connected to an endodontic instrument. The other side is connected to the patient’s body, either by a contact to the patient’s lip or by an electrode held in the patient’s hand. The electrical circuit is complete when the endodontic instrument is advanced apically inside the root canal until it touches periodontal tissue. The display on the apex locator indicates that the apical area has been reached. Many in vitro accuracy studies were conducted on models using an extracted tooth in an electrolyte to simulate clinical conditions. The ideal conditions in in vitro testing may give accuracy results higher than those obtainable in clinical practice (17-25).

Therefore the main aim of our study was to test the efficacy of ROOT ZX (J. Morita Mfg Corp., Kyoto, Japan), a third-generation apex locator based on “ratio method” that uses dual frequency and comparative impedance principles (26,27). The Root ZX is mainly based on detecting the change in electrical capacitance that occurs near the apical constriction. Some of the advantages of the Root ZX are that it requires no adjustment or calibration and can be used when the canal is filled with strong electrolyte or when the canal is “empty” and moist (26).

## Material and Methods

Sixty, straight, single-rooted permanent human teeth with mature apices were selected for this study. The teeth were cleaned of calculus, soft tissues, and debris with hand instrumentation and stored in distilled water until used. The type I canal configuration was confirmed by using digital radiograph (Gendex, Dentsply) in mesiodistal and labiolingual planes. Teeth with resorption, curvatures, open apices, or radiographically invisible canals were excluded from the study.

The teeth were decoronated at the level of cementoenamel junction with a diamond disc to allow access to the root canal and to provide a stable reference for all measurements. The coronal portion of each canal was preflared using sequential Gates Glidden drills #4, #3, and #2 (Mani Inc., Japan), irrigated with saline and pulp extirpated with a barbed broach (Spirocolorinox, Dentsply).

- Measurement of actual working length 

Teeth were numbered 1–60 and the actual canal length (AL) was determined by introducing a size 10 or 15 k-file (Mani Inc., Japan) into the canal until its tip emerged through the major apical foramen. 3.5x magnification loops were used during this procedure for enhanced visibility. The long axis of the tooth was placed perpendicular to the line of sight and the tip of the file will be positioned tangential to the major apical foramen (28,29). After carefully adjusting the silicone stopper to the reference point, the file was withdrawn from the root canal, and the distance between the file tip and silicone stopper was measured with a digital caliper (Mitutoyo Co., Japan) and the reading was noted down.

To simulate the periodontium, this study used the in vitro model as designed by Donnelly (30). A polystyrene specimen bottle (40 ml) was filled with warmed gelatin solution and refrigerated for 2 h to allow gelatin to set. The apical two-third of the root was embedded in gelatin, and the tooth was stabilized to the lid of a container with auto-polymerizing resin as described by Higa et al (29). The lip electrode was also placed in gelatin through another opening in the lid.

- Working model for electronic working length determination 

The irrigants tested will be: 0.9% saline, 2.5% sodium hypochlorite (NaOCl) and 0.2% chlorhexidine gluconate (CHX) (*Curasept, Italy*). The irrigant to be tested was introduced into the canal with a 23-guage needle.

The EAL tested in this experiment was: Root ZX (J. Morita Mfg Corp., Kyoto, Japan). It was used according to manufacturer’s instructions (31). Depending on the size of the canal, #15 or #20 K-file (Mani Inc., Japan), was attached to the file holder and introduced into the canal.

The meter’s 0.5 mm reading was set between the “APEX” and “1” (factory setting) as indicated by a flashing bar and will be used for electronic measurements. The file was gently inserted into the root canal until the “APEX” signal will be displayed. The file was then gently retracted until the display showed a flashing image of the root canal and a flashing bar between APEX and 1 (0.5 reading). The silicone stopper on the file will be carefully adjusted to a reference point, and the file was withdrawn to measure the distance between the silicone stopper and the file tip and noted down. This was recorded as the electronically measured canal length (EL).

The electronically measured Working length (EL) was compared with the actual canal (AL) length measured conventionally and scores were attributed to the resulting values (32) ( Table 1).

**Table 1:**
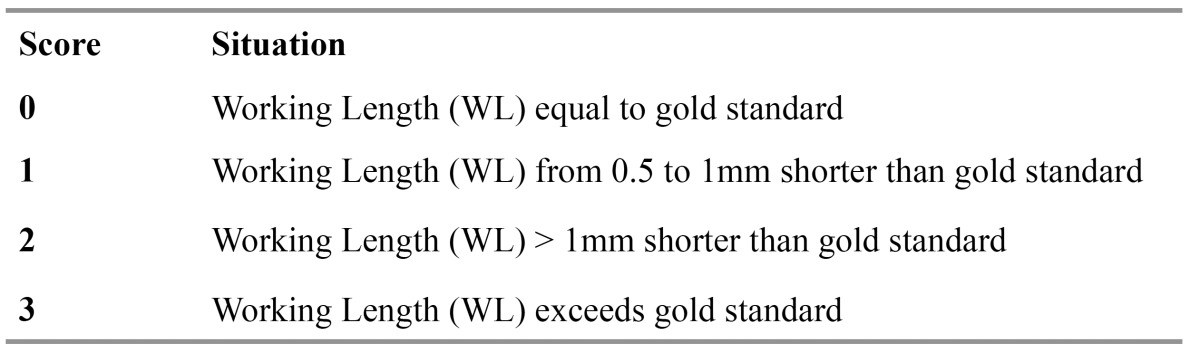
Comparation between the electronically measured Working length (EL) and the actual canal (AL).

The canal length was assessed for each tooth with individual irrigants. To prevent cross-contamination: (a) fresh gelatin was used for the individual irrigant, and (b) the root canals was irrigated with ethanol and dried with paper points. The results obtained (in millimeters) were recorded. The difference between the median of electronically measured length (EL) and the AL was calculated for each tooth in the presence of all irrigating solutions. The resulting difference in working length was noted down.

A paired t-test was employed to statistically analyze the significance of mean difference between EL and AL. One-way ANOVA was also employed along with Welch & Brown-Forsythe analysis to assess the significance of difference among various irrigants in their estimations of the canal length. Level of significance was set at P <0.05. The analysis was performed with Statistical and Presentational System Software (SPSS 20.0, SPSS Inc, Chicago, IL).

## Results

 Table 2,  Table 3 and  Table 4 shows the actual working length obtained with conventional method and the electronically measured working length obtained with Root Zx with different group of irrigants (*0.9% Saline, 2.5% NaOCl & 0.2% Chlorohexidine*). It also indicates the score (*as mentioned in table 1*) and the difference in working length obtained per tooth.

**Table 2:**
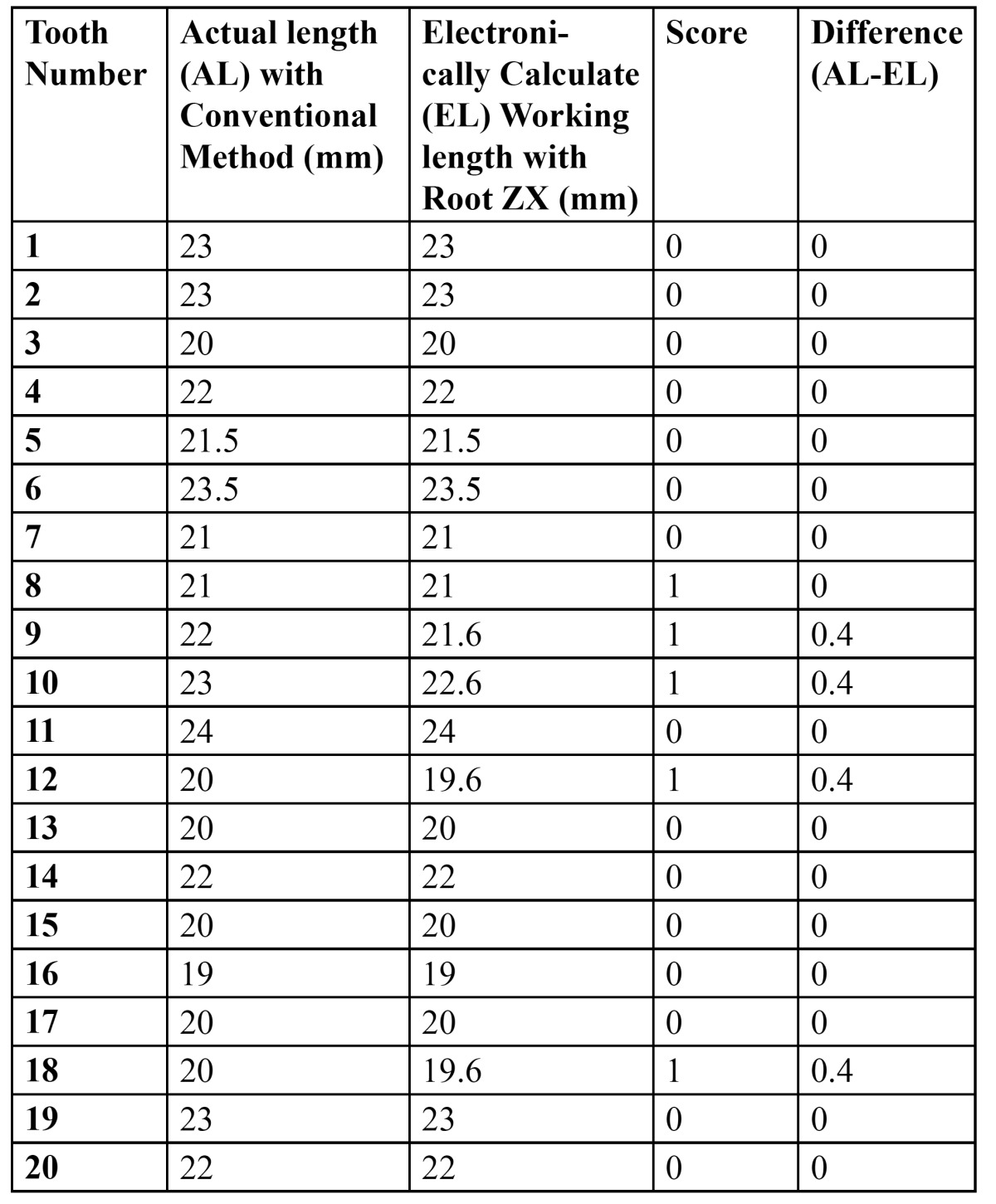
Group 1 (0.9% Saline as irrigant).

**Table 3:**
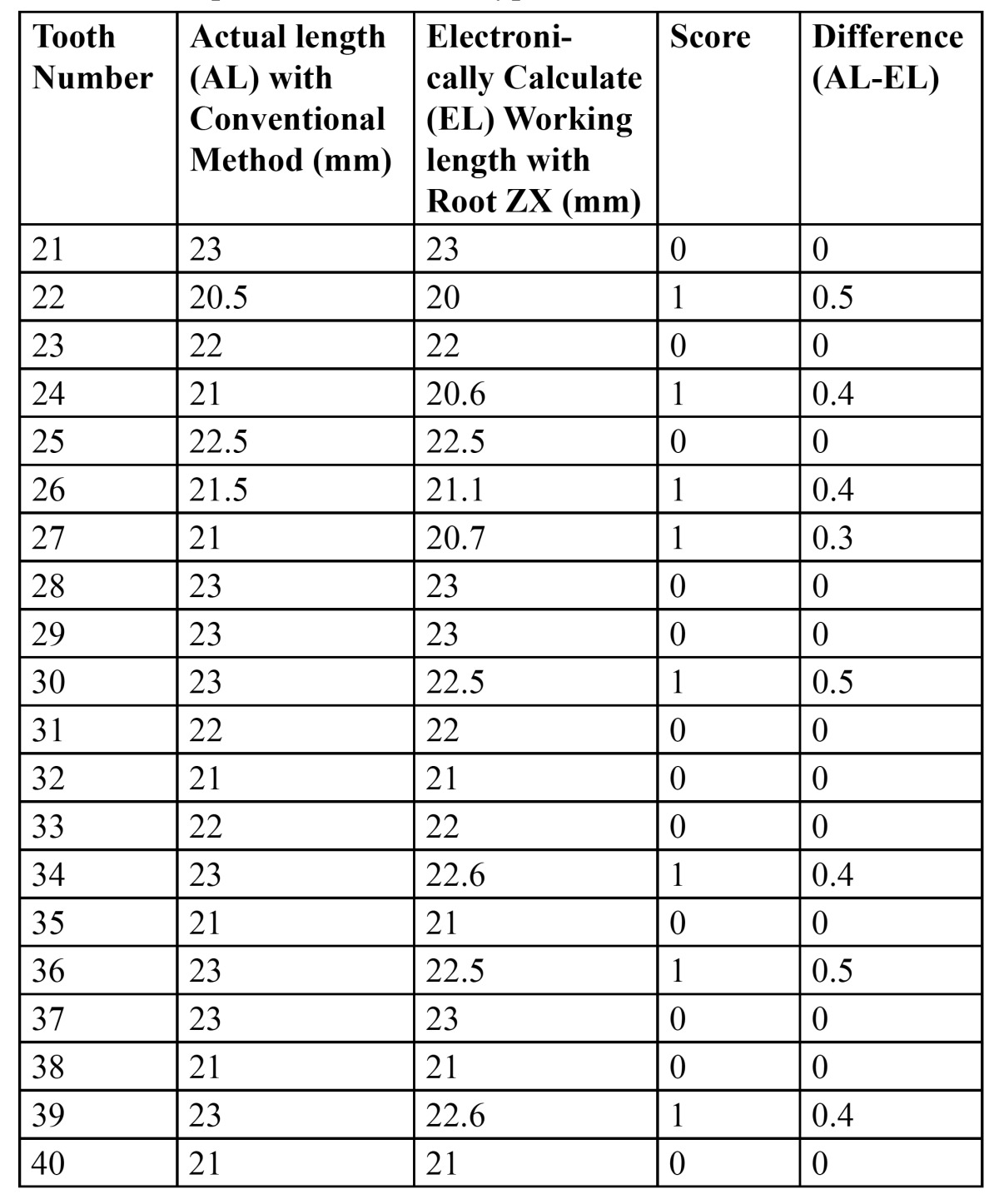
Group 1 (2.5% Sodium Hypochlorite - NaOCl).

**Table 4:**
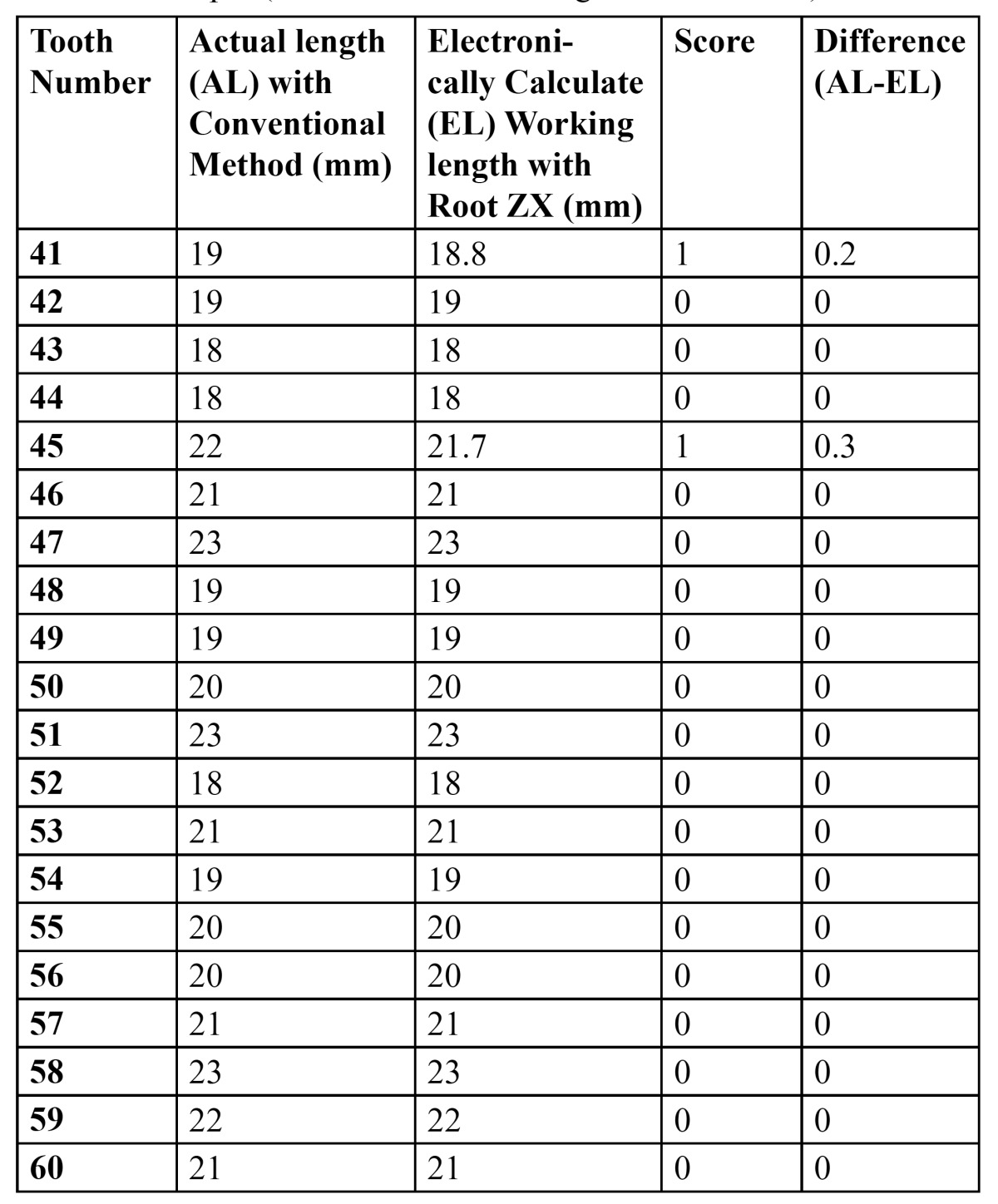
Group 1 (0.2% Chlorohexidine gluconate - CHX)

The mean values of actual canal length and electronically measured working length with Root ZX along with their differences for all three groups are given in  Table 5.

**Table 5:**
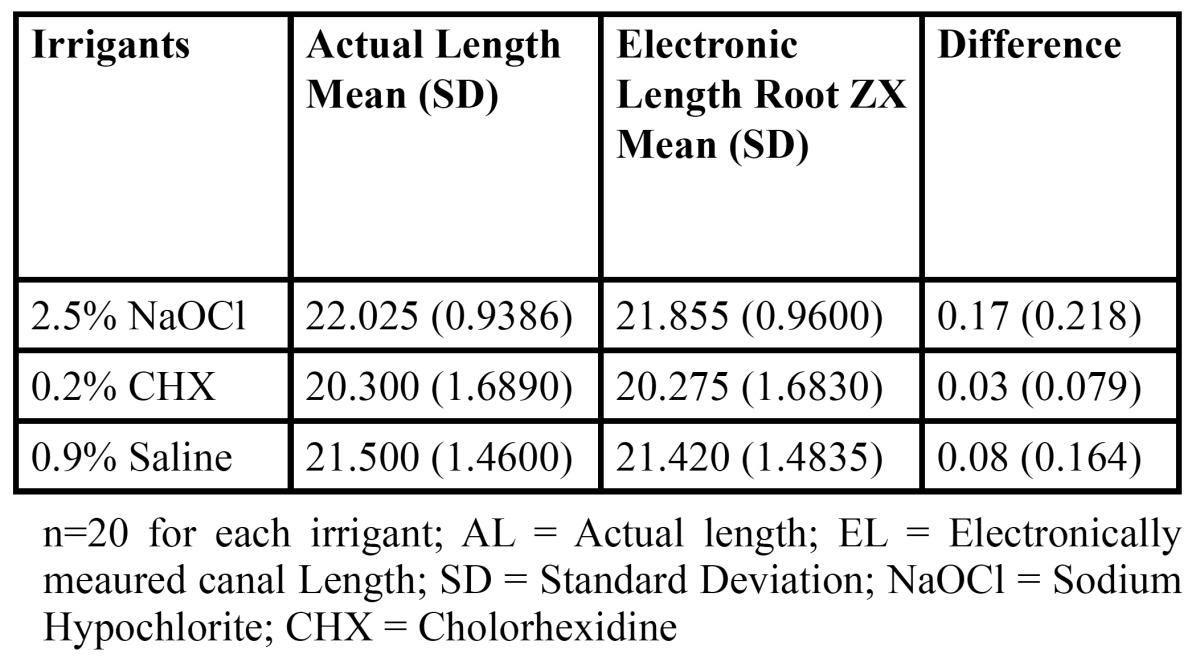
Mean (SD) of AL and EL measurements with Root ZX in the presence of various irrigants.

 Table 6 shows that the working length differences obtained by different groups of irrigants is statistically significant as P value = 0.024. Robust test of equality of means was also employed and the outcome of Welch and Brown-Forsythe analysis is shown in  Table 7 where P value is 0.022 and 0.026 respectively. Therefore proving further statistical significance.

**Table 6:**
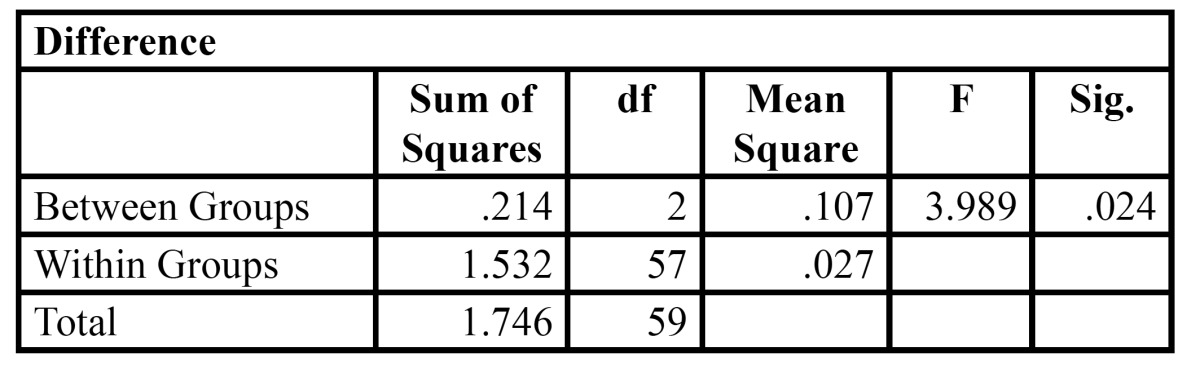
Anova.

**Table 7:**
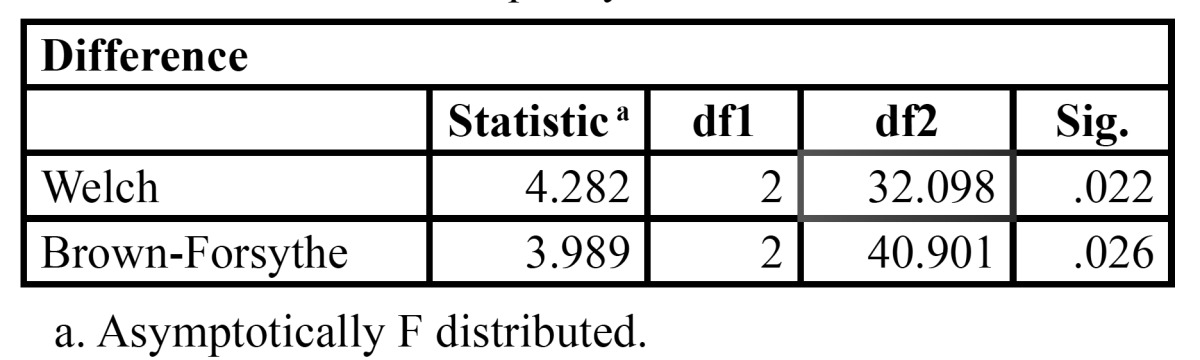
Robust Test of equality of means.

Our study indicated that the differences in working length obtained with 2.5% NaOCl were significantly larger than those obtained with 0.2% Chlorohexidine and 0.9% Saline. Although statistically significant differences existed between the irrigants, the majority of the readings were within the acceptable range of ±0.5 mm for Root ZX

The results indicate that root ZX was accurate within ±0.5mm 100% of the time with all the three test irrigants.

## Discussion

The first-generation EALs were resistance-based and the second-generation EALs were impedance-based apex locators (33). The main shortcomings of these EALs included poor accuracy in the presence of fluids and pulp tissue, and the need for calibration (34). The frequency-based third-generation EALs have more powerful microprocessors and are able to process mathematical quotient and algorithm calculations required to give accurate readings (35). Root ZX (J. Morita Mfg Corp., Kyoto, Japan) is a third-generation EAL that uses dual frequency and comparative impedance principle is based on the “ratio method” for measuring canal length. This method simultaneously measures the impedance values at two frequencies (8 and 0.4 kHz) and calculates a quotient of impedances. This quotient is expressed as a position of the file in the canal. Root ZX requires no calibration, and can be used when the canal is filled with a strong electrolyte (36).

The present study used an in vitro model as described by Donnelly (30) to obtain accurate measurements. The advantages of the model were its simplicity, ease of use and the ability to have strict control over the tested experimental condition. A disadvantage of the model is its inability to fully simulate in vivo conditions. Huang supported the use of in vitro models for evaluation of electronic apex locators (37). According to him, when tip of the file passes through narrow apical foramen, the physical properties of the foramen itself produce the electrical resistance gradient.

The use of irrigating solutions is an important aspect of endodontic treatment. Several studies using advanced techniques such as micro-computed tomography (CT) scanning have demonstrated that proportionally large areas of the main root-canal wall remain untouched by the instruments (38). Sodium hypochlorite (NaOCl) is the most popular irrigating solution. NaOCl ionizes in water into Na and the hypochlorite ion, OCl, establishing an equilibrium with hypochlorous acid (HOCl). At acidic and neutral pH, chlorine exists predominantly as HOCl, whereas at high pH of 9 and above, OCl predominates (39). Hypochlorous acid is responsible for the antibacterial activity. NaOCl is commonly used in concentrations between 0.5% and 6%. It is a potent antimicrobial agent, killing most bacteria instantly on direct contact. It also effectively dissolves pulpal remnants and collagen, the main organic components of dentin. Hypochlorite is the only root-canal irrigant of those in general use that dissolves necrotic and vital organic tissue. It is difficult to imagine successful irrigation of the root canal without hypochlorite.

Chlorhexidine gluconate (CHX) is widely used in disinfection in dentistry because of its good antimicrobial activity (40). CHX is marketed as a water-based solution and as a gel (with Natrosol). Some studies have indicated that the CHX gel has a slightly better performance than the CHX liquid but the reasons for possible differences are not known (41). CHX solutions in concentrations of 0.2–2% are considered toxicologically safe (42). However, there is paucity of research regarding the accuracy of EAL in presence of Chlorhexidine (43). It is imperative that the clinician should be confident of the fact that irrigating solution is not effecting the accurateness of the apex locator.

The results of this study showed that, Root ZX is 100% accurate within 0.5 mm from the apical foramen. The ± 0.5 mm to the foramen range has been considered as the strictest acceptable range (44). Thus, measurements attained within this tolerance are considered highly accurate. The results of measurements which are within this range are considered accurate. According to this study, statistically significant difference was found when measurements were done in canals irrigated with 2.5% sodium hypochlorite. However these were within the clinically acceptable range of within 0.5mm. The possible reason for this variation could be the higher electrical conductivity of sodium hypochlorite.

The use of electronic devices to determine working length has gained increasing popularity in recent years (45). The use of apex locators is very useful in scenarios like anatomical limitations for example zygomatic arch or maxillary sinus where at times. Also at times distortion of radiographs limits the accuracy of working length determination.

The results of the studies confirm that EALs can accurately determine the canal length within ±0.5 mm from the apical constriction (46). The Root ZX has become a benchmark to which other devices are compared with. The results of this study are comparable to previously studies done (47-48).

This study shows that Root ZX can reliably be used for determining the position of the apical foramen in the presence of above mentioned irrigating solutions.

## Conclusion

The results of the present study confirm that EALs can accurately determine the root canal length within ±0.5 mm from the apical constriction. Therefore it is clinically safe and accurate to use Electronic apex locator with all the three mentioned irrigating solutions in this study.
